# Real-world data on metabolic effects of PCSK9 inhibitors in a tertiary care center in patients with and without diabetes mellitus

**DOI:** 10.1186/s12933-021-01283-w

**Published:** 2021-04-24

**Authors:** Laurenz T. Fischer, Daniel A. Hochfellner, Lisa Knoll, Tina Pöttler, Julia K. Mader, Felix Aberer

**Affiliations:** Division of Endocrinology and Diabetology, Medical University of Graz, Auenbruggerplatz 15, 8036 Graz, Austria

**Keywords:** PCSK9 inhibitor therapy, Lipid lowering therapy, Real world data

## Abstract

**Background:**

The lipid-lowering and positive cardiovascular effect of proprotein convertase subtilisin kexin type 9 (PCSK9) inhibitors was shown in several studies, hence, they are more widely used in the lipid-lowering management of individuals with high cardiovascular risk. As real-world data are still scarce, specifically in patients with type 2 diabetes (T2D), the aim of this retrospective analysis was to investigate the efficacy of PCSK9 inhibitors in lowering low-density lipoprotein cholesterol (LDL-C) in an outpatient clinic of a tertiary care center in routine care.

**Methods:**

A retrospective analysis of data extracted from the electronic patient record was performed. Patients who were routinely prescribed with PCSK9 inhibitor therapy (alirocumab or evolocumab) during the years 2016 and 2019 were included in the analysis. Characteristics of the patient population, the effects on LDL-C and HbA1c levels as well as subsequent cardiovascular events were assessed over an observation period of 18 months.

**Results:**

We identified 237 patients treated with PCSK9 inhibitors between January 2016 and September 2019. Almost all patients (97.5%) received PCSK9 inhibitors for secondary prevention. 26.2% of the population had a concomitant diabetes diagnosis. Intolerance to statins (83.1%), ezetimibe (44.7%) or both agents (42.6%) was reported frequently. Three months after initiation of PCSK9 inhibitor therapy, 61.2% of the patients achieved LDL-C levels < 70 mg/dl, and 44.1% LDL-C levels < 55 mg/dl. The median LDL-C was lowered from 141 mg/dl at baseline, to 60 mg/dl after 3 months and 66 mg/dl after 12 months indicating a reduction of LDL-C as follows: 57.5% after 3 months and 53.6% after 12 months. After 3 months of observation, target achievement of LDL-C was higher in patients with T2D compared to non-diabetes patients; < 55 mg/dl: 51% vs. 41.5%; < 70 mg/dl 69.4 vs. 58.5%. After 12 months even more pronounced target LDL achievement in T2D was demonstrated < 55 mg/dl: 58.8% vs. 30.1%; < 70 mg/dl 70.6 vs. 49.6%. Patients with insufficiently controlled T2D (HbA1c > 54 mmol/mol) had a higher reduction in LDL-C but still were more likely to subsequent cardiovascular events.

**Conclusions:**

Significant reductions in LDL-C and a high percentage of patients achieving recommended treatment targets were observed. The percentage of patients with T2D meeting recommended LDL-C targets was higher than in those without T2D. Still some patients did not achieve LDL-C levels as recommended in current guidelines. Special attention to the characteristics of these patients is required in the future to enable achievement of treatment goals and avoid adverse cardiovascular outcomes.

**Supplementary Information:**

The online version contains supplementary material available at 10.1186/s12933-021-01283-w.

## Background

Low-density lipoprotein cholesterol (LDL-C) is a well-established risk factor in the development of atherosclerotic cardiovascular disease (ASCVD). Several studies have shown unequivocal evidence that high levels of LDL-C have an unfavorable effect on ASCVD and contribute to cardiovascular death [1–3]. At present, statins are in addition to lifestyle interventions the first-line therapy for LDL-lowering, in addition to lifestyle interventions, in most patients. Further pharmacological lipid-lowering options include combinations of ezetimibe, bile acid sequestrants, fibrates and proprotein convertase subtilisin kexin type 9 (PCSK9) inhibitors [4].

Both, the FOURIER trial on evolocumab and the ODYSSEY OUTCOMES trial on alirocumab showed that PCSK9 inhibitors were not only capable to significantly lower LDL-C levels, but also result in a substantial reduction of the cardiovascular event rate without relevant risk of adverse events [5, 6]. As recommended in the guidelines for the management of dyslipidemias by the European Society of Cardiology (ESC) and European Atherosclerosis Society (EAS), treatment with a PCSK9 inhibitor is indicated for secondary prevention to reduce plasma LDL-C in very-high risk patients who do not achieve their target LDL-C or even for primary prevention in particular very-high risk patients as those with familial hypercholesterolemia (FH) who do not achieve their LDL-C goal despite maximal tolerated therapy with statins and ezetimibe [4]. In 2015, the PCSK9 inhibitors alirocumab and evolocumab were approved in the European Union by the European Medicines Agency.

Patients with diabetes represent a particular risk group for cardiovascular disease and consecutive events. Evidence gained from randomized controlled trials using PCSK9 inhibitors in secondary prevention suggests a similar efficacy on LDL-C reduction and clinical benefit in patients with diabetes mellitus compared to those without diabetes [7, 8]. However, hardly any observational trials that confirmed these results when investigated during real-world conditions are available.

Since patients who participate in clinical trials are usually seen on a regular and rather frequent basis throughout the course of the study, are provided free of charge with study medication and adherence to medication is closely monitored which can be subsumed as study effect, there might be differences in routine care that are worth investigating. In addition, most of the available real-world studies on PCSK9 inhibitors only investigated small groups of patients and did not specifically focus on patients with diabetes mellitus.

Previous research suggested a potential diabetogenic effect by using statins in the treatment of hyperlipidemia [9]. Also, genetic studies demonstrated that specific PCSK9 variants might increase the risk for the development of diabetes mellitus type 2 (T2D) [10], however this finding could not be evidently verified in randomized controlled trials (RCTs) when PCSK9 inhibitors were exogenously administered [11].

The aim of this retrospective analysis was to investigate the effect of PCSK9 inhibitor therapy on markers of lipid metabolism, to determine patient characteristics, to assess the indications for their use and to evaluate the tolerance of PCSK9 inhibitor therapy in patients with and without T2D in a routine care-setting of an outpatient clinic in a tertiary care center. In addition, we intended to examine potential influence of PCSK9 inhibitor therapy on glycemic control assessed by HbA1c and evaluated whether there were differences in LDL-C reduction efficacy in those with and without T2D. Furthermore, the population was screened for subsequent cardiovascular events within an observation period of 18 months after initiation of PCSK9 inhibitor therapy.

## Material and methods

### Patients and outcome measures

This study was a retrospective data analysis approved by the Medical University of Graz, Austria (EK number 32-018 ex 19/20) and included cardiovascular high-risk patients who were prescribed with PCSK9 inhibitor therapy within routine conditions, considering the national reimbursement criteria (LDL > 100 mg/dl despite maximal tolerated statin/ezetimibe therapy, well controlled hypertension, HbA1c < 64 mmol/mol, having received nutritional advice by a dietologist and intensive motivation to stop smoking). Data of the electronic patient records of the University hospital were screened for eligible patients from January 2016 to September 2019. Electronic records of adult patients with current or past PCSK9 inhibitor treatment in routine care at the outpatient clinic of the Division of Endocrinology and Diabetology were searched and included in the analysis if they met the inclusion criteria. Inclusion criteria were as follows: age > 18 years, treatment with locally available PCSK9 inhibitors (alirocumab 75 or 150 mg, or evolocumab 140 mg) in routine care, available laboratory reports on LDL-C levels at first prescription and LDL-C during a follow-up period longer than 3 months.

In eligible patients the following parameters were drawn from the electronic patient record: age, sex, lipid-lowering therapy at baseline (i.e. statins, ezetimibe, fibrates), cause of prescription of PCSK9 inhibitor (intolerance to lipid-lowering medications at baseline, failure to achieve individual LDL-C levels; primary or secondary prevention as indication), type of PCSK9 inhibitor (i.e. alirocumab or evolocumab), PCSK9 inhibitor therapy adjustments (agent, discontinuation). Baseline macrovascular (coronary heart disease, stroke, transient ischemic attack, peripheral artery disease, carotid artery disease) and microvascular (retinopathy, nephropathy) comorbidities, previous cardiovascular interventions (i.e. percutaneous coronary revascularization or coronary artery bypass grafting) and further cardiovascular risk factors (i.e. smoker status, hypertension, diabetes mellitus) were assessed. The following laboratory parameters were extracted: LDL-C, high-density lipoprotein cholesterol (HDL-C), triglycerides (TG), total cholesterol, lipoprotein (a) (Lp[a]), and HbA1c at baseline and during PCSK9 inhibitor therapy for up to 18 months whenever collected in routine care. Number of patients who were lost to follow-up (missing documentation regarding PCSK9 inhibitor therapy ≥ 1 year) or patients who discontinued therapy (i.e. adverse drug effects towards PCSK9; noncompliance) were also determined. Baseline was defined as the day of the first PCSK9 inhibitor application. Patients enrolled were followed up for subsequent cardiovascular events within the first 18 months after PCSK9 inhibitor initiation. MACE was defined as occurrence of non-fatal myocardial infarction or stroke or cardiovascular death. All-cause cardiovascular events included the following: any acute coronary syndrome, scheduled coronary intervention, aortic dissection, any cerebral ischemic event (transitory ischemic attack [TIA] or stroke), any peripheral vascular intervention or diagnosis/worsening of peripheral artery disease, newly diagnosed carotid artery disease, surgical carotid intervention, necessity of hemodialysis or acute decompensated heart failure.

### Statistical methods

The follow-up of LDL-C, HDL-C, TG, Lp(a) and HbA1c were analyzed at months 3, 6, 9 and 12, and up to 18 months from baseline, including data from the most current measurement after the defined time points. A last observation carried forward analysis was performed for laboratory parameters, imputing data of the latest measurement of maximum 3 months prior. If PCSK9 inhibitor therapy was discontinued, further observations were not included in the analyses.

Wilcoxon signed-rank test was used to compare parameters between baseline and follow-up time points within a subgroup, Mann–Whitney-U test was used to evaluate between-group differences of two different subgroups. Kruskal–Wallis test was used to analyze differences of LDL-C levels and LDL-C reduction across subgroups of concomitant lipid-lowering therapy. Univariate Cox‐regression analysis was used to calculate hazard ratios for events of patients with T2D compared to patients without T2D. P-values of ≤ 0.05 were considered statistically significant. Statistical analysis was performed with IBM SPSS Statistics version 26.

## Results

### Patient characteristics

We identified 237 eligible patients (47.7% female) who received alirocumab or evolocumab (104 and 133 patients) of whom the median age was 65.2 years (interquartile range [IQR] 57.8–71.5). Almost all patients (97.5%; n = 231) received PCSK9 inhibitors for secondary prevention. Apart from hypercholesterolemia, more than half of the study population (53.6%) had two or more additional cardiovascular risk factors (arterial hypertension, chronic kidney disease, Age ≥ 65 years, diabetes mellitus, smoker). Medical history included coronary heart disease (74.7%), at least one percutaneous coronary intervention (46.4%), coronary artery bypass graft (CABG) (16%), stroke or transient ischemic attack (13.5%), carotid artery stenosis (30.8%), peripheral artery disease (18.1%), and chronic kidney disease (9.7%). Any type of familial hypercholesterolemia (mostly diagnosed using clinical criteria, no one identified with homozygous FH) was present in 21.5% of the patients. Diabetes mellitus was present in 62 patients of whom the majority had type 2 diabetes (T2D) (n = 54). Further relevant comorbidities at baseline were: arterial hypertension (68.8%) and active smoker status (5.9%).

Intolerance to other lipid-lowering agents was frequently present at baseline: 83.1% reported intolerance to at least one statin, 44.7% reported side-effects to ezetimibe. 42.6% indicated statin and ezetimibe intolerance. The full characteristics of the patient cohort are listed in Table 1. The more detailed summary of cardiovascular risk factor profile is shown in Additional file 1: Table S1. Due to the retrospective design of this study, we were not able to ascertain laboratory data at the predefined time-points from all patients (i.e. every 3 months after therapy initiation) due to loss to follow-up, discontinuation of treatment or infrequent outpatient clinic visits. Ten patients (4.2%) discontinued PCSK9 inhibitor therapy (five after 3 months from baseline, one after 6 months, one after 9 months, three after 12 months) and were not further included in the analysis after discontinuation. 27 patients (11.4%) were lost to follow-up (missing documentation regarding PCSK9 inhibitor therapy ≥ 1 year).

**Table 1:** Baseline characteristics overall as well as per PCSK9 inhibitor used and diabetes status

Characteristics	Total (n = 237)	Alirocumab (n = 104)	Evolocumab (n = 133)	Without diabetes mellitus (n = 175)	With diabetes mellitus type 2 (n = 54)
Age (years)	65.2 (57.8–71.5)	65.0 (57.8–71.5)	65.4 (57.9–71.6)	65.0 (57.8–70.8)	68.9 (63.1–73.8)
Female sex n (%)	113 (47.7)	53 (51.0)	60 (45.1)	88 (50.3)	22 (40.7)
Indication for PCSK9 inhibitor treatment n (%)					
Primary prevention	6 (2.5)	4 (3.8)	2 (1.5)	4 (2.3)	2 (3.7)
Secondary prevention	231 (97.5)	100 (96.2)	131 (98.5)	171 (97.7)	52 (96.3)
Medical history n (%)					
Coronary heart disease	177 (74.7)	80 (76.9)	97 (72.9)	134 (76.6)	38 (70.4)
Percutaneous coronary intervention	110 (46.4)	49 (47.1)	61 (45.9)	86 (49.1)	21 (38.9)
Coronary artery bypass graft	38 (16.0)	16 (15.4)	22 (16.5)	27 (15.4)	8 (14.8)
Stroke or transient ischemic attack	32 (13.5)	12 (11.5)	20 (15.0)	24 (13.7)	8 (14.8)
Carotid artery disease	73 (30.8)	37 (35.6)	36 (27.1)	51 (29.1)	20 (37.0)
Peripheral artery disease	43 (18.1)	15 (14.4)	28 (21.1)	25 (14.3)	15 (27.8)
Arterial hypertension	163 (68.8)	74 (71.2)	89 (66.9)	112 (64.0)	46 (85.2)
Familial hypercholesterolemia (heterozygous)	51 (21.5)	19 (18.3)	32 (24.1)	41 (23.4)	8 (14.8)
Retinopathy	10 (4.2)	5 (4.8)	5 (3.8)	4 (2.3)	3 (5.6)
Chronic kidney disease	23 (9.7)	6 (5.8)	17 (12.8)	10 (5.7)	12 (22.2)
Current tobacco smoker	14 (5.9)	9 (8.7)	5 (3.8)	12 (6.9)	1 (1.9)
Diabetes mellitus any type	62 (26.2)	25 (24.0)	37 (27.8)	–	–
Diabetes mellitus type 1	6 (2.5)	2 (1.9)	4 (3.0)	–	–
Diabetes mellitus type 2	54 (22.8)	22 (21.2)	32 (24.1)	–	54 (100)
Other types of diabetes	2 (0.8)	1 (1.0)	1 (0.8)	–	–
Intolerances/side effects to lipid-lowering medication n (%)					
Statins	197 (83.1)	83 (79.8)	114 (85.7)	147 (84.0)	43 (79.6)
Ezetimibe	106 (44.7)	36 (34.6)	70 (52.6)	76 (43.4)	28 (51.9)
Statins and ezetimibe	101 (42.6)	34 (32.7)	67 (50.4)	74 (42.3)	25 (46.3)

Patients with type 1 diabetes were not separately analysed due to the small number (n = 4). Data are median (interquartile range) or number (%)

### Laboratory parameters and concomitant medication at baseline

At baseline, 29.5% of the overall cohort were on statin therapy (mostly rosuvastatin or atorvastatin), 39.7% on ezetimibe and 2.5% on fibrates. 48.9% did not receive lipid-lowering medication (i.e. no statins, ezetimibe or fibrates) at that time. Concomitant lipid-lowering therapy of patients with T2D was comparable to patients without diabetes mellitus. There was only a slight difference in a higher usage of fibrates in the group of patients with diabetes mellitus. Median LDL-C at baseline was 141 mg/dl (117–188), distribution of alirocumab and evolocumab use was 44 and 56%. Median HbA1c in the T2D population was 52 mmol/mol (48–57 mmol/mol; 6.9% [6.5–7.4]%). Further laboratory parameters and concomitant medication at baseline according to the prescribed PCSK9 inhibitor and diabetes status are shown in Table 2.

**Table 2:** Laboratory parameters, concomitant lipid-lowering and anti-hyperglycemic therapy at baseline according to prescribed PCSK9 inhibitor and diabetes status

Characteristics	Total (n = 237)	Alirocumab (n = 104)	Evolocumab (n = 133)	Without diabetes mellitus (n = 175)	With diabetes mellitus type 2 (n = 54)
LDL-C (mg/dl)	141 (117–188) n = 237	135 (114–181) n = 104	149 (118–191) n = 133	141 (117–188) n = 175	135 (110–178) n = 54
Total cholesterol (mg/dl)	229 (198–268) n = 216	210 (190–259) n = 94	240 (202–272) n = 122	230 (200–268) n = 163	216 (194–261) n = 47
HDL-C (mg/dl)	54 (45–65) n = 226	52 (44–63) n = 100	55 (46–67) n = 126	54 (46–67) n = 169	49 (42–60) n = 49
Triglycerides (mg/dl)	138 (99–215) n = 227	138 (102–255) n = 102	138 (97–197) n = 138	124 (97–199) n = 167	185 (134–249) n = 52
Lp(a) (mg/dl)	65 (25–101) n = 47	87 (71–114) n = 13	55 (18–90) n = 34	65 (23–114) n = 37	67 (46–89) n = 10
HbA1c (mmol/mol)	41 (37–49) n = 139	40 (37–49) n = 61	43 (37–50) n = 78	38 (36–40) n = 81	52 (48–57) n = 51
Statins (n; %)	70 (29.5)	38 (36.5)	32 (24.1)	52 (29.7)	16 (29.6)
Fluvastatin	3 (1.3)	2 (1.9)	1 (0.8)	2 (1.1)	1 (1.9)
Pravastatin	0 (0)	0 (0)	0 (0)	0 (0)	0 (0)
Simvastatin	5 (2.1)	1 (1.0)	4 (3.0)	5 (2.9)	0 (0)
Rosuvastatin	29 (12.2)	15 (14.4)	14 (10.5)	22 (12.6)	5 (9.3)
Atorvastatin	35 (14.8)	21 (20.2)	14 (10.5)	25 (14.3)	10 (18.5)
Ezetimibe	94 (39.7)	46 (44.2)	48 (36.1)	67 (38.3)	22 (40.7)
Fibrates	6 (2.5)	2 (1.9)	4 (3.0)	2 (1.1)	4 (7.4)
No lipid-lowering medication	116 (48.9)	44 (42.3)	72 (54.1)	89 (50.9)	24 (44.4)
Antidiabetic treatment					
Metformin	24 (10.1)	13 (12.5)	11 (8.3)	0 (0)	23 (42.6)
SGLT2 inhibitors	5 (2.1)	2 (1.9)	3 (2.3)	0 (0)	4 (7.4)
DPP-4 inhibitors	14 (5.9)	7 (6.7)	7 (5.3)	0 (0)	14 (25.9)
GLP-1 receptor agonists	4 (1.7)	0 (0)	4 (3.0)	0 (0)	4 (7.4)
Sulfonylureas	1 (0.4)	0 (0)	1 (0.8)	0 (0)	1 (1.9)
Pioglitazone	2 (0.8)	1 (1.0)	1 (0.8)	0 (0)	2 (3.7)
Insulin therapy	23 (9.7)	9 (8.7)	14 (10.5)	0 (0)	16 (29.6)
Diet only	49 (20.7)	22 (21.2)	27 (20.3)	0 (0)	14 (25.9)

Data are median (interquartile range) or number (%)

*SGLT2* sodium-glucose co-transporter 2, *GLP-1* Glucagon-like peptide 1, *DPP-4* dipeptidyl-peptidase 4

### Effect on LDL-C levels

**Fig. 1:**
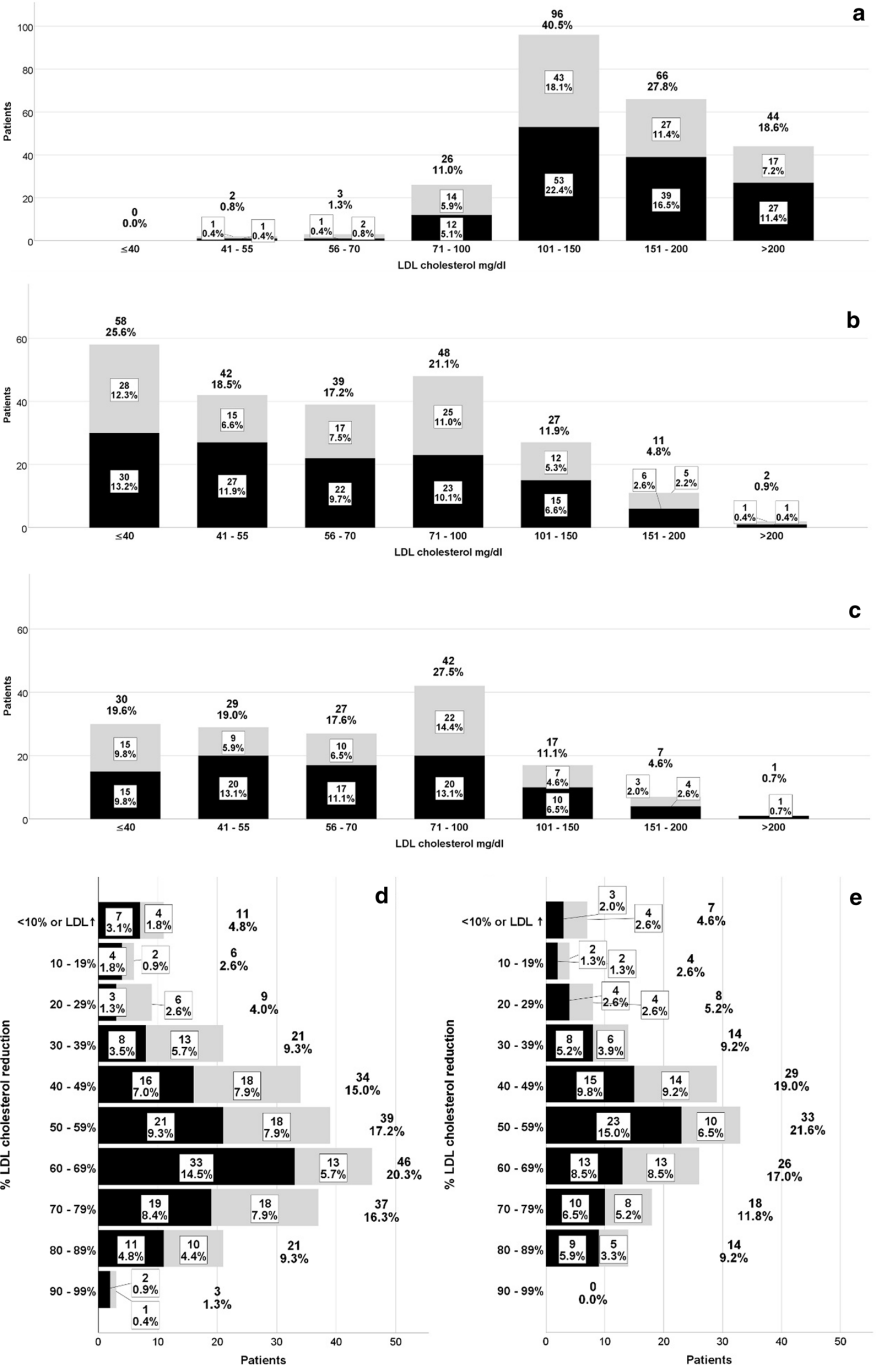
LDL-C levels and percentage of LDL-C reduction over time; data are number of patients (n) and percentage (%); grey bar = alirocumab, black bar = evolocumab. **a** Baseline LDL-C levels (n = 237), **b** LDL-C levels at month 3 (n = 227), **c** LDL-C levels at month 12 (n = 153); **d** % LDL-C reduction from baseline to month 3 (n = 227) **e** % LDL-C reduction from baseline to month 12 (n = 153)

Median baseline LDL-C of the total population at baseline was 141 mg/dl and decreased to 60, 59, 61 and 66 mg/dl after 3, 6, 9 and 12 months of observation, respectively. During the course of treatment, a substantial proportion of patients achieved LDL-C levels < 70 mg/dl (61.2% after 3 months and 56.2% after 12 months) or < 55 mg/dl (44.1% after 3 months and 38.6% after 12 months). LDL-C remained above 100 mg/dl in 17.6% at month 3 months and in 16.3% at month 12. LDL-C reduction > 50% was achieved in 64.3% and 59.5% at month 3 and 12, respectively (Fig. 1). Additional file 1: Table S2 shows the LDL-reduction over time according to the PCSK9 inhibitor used.

### LDL-C reduction according to diabetes status and quality of glycemic control

**Fig. 2:**
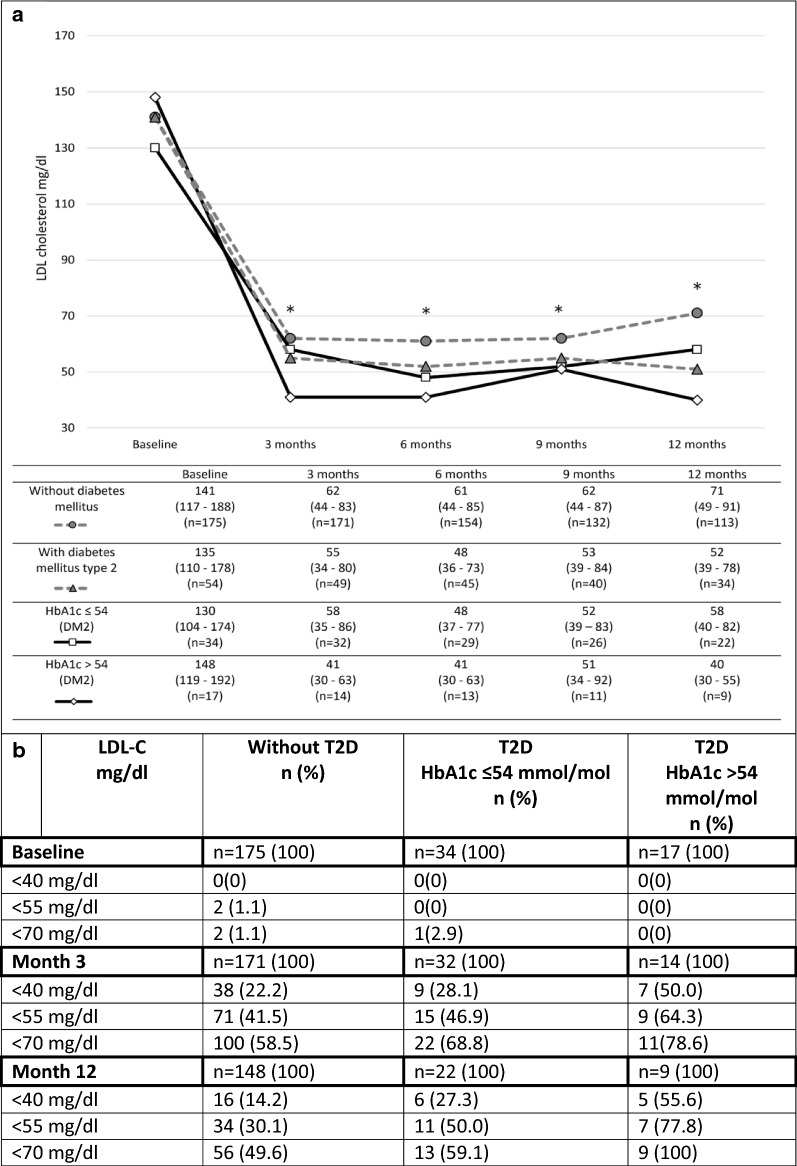
**a** LDL-C levels of patients without and with T2D 2. **b** Number and percentage of achievement of treatment targets according to diabetes status. Data are median (interquartile range). *Indicates statistical significance when compared to baseline LDL-C levels (for all groups investigated)

**Fig. 3:**
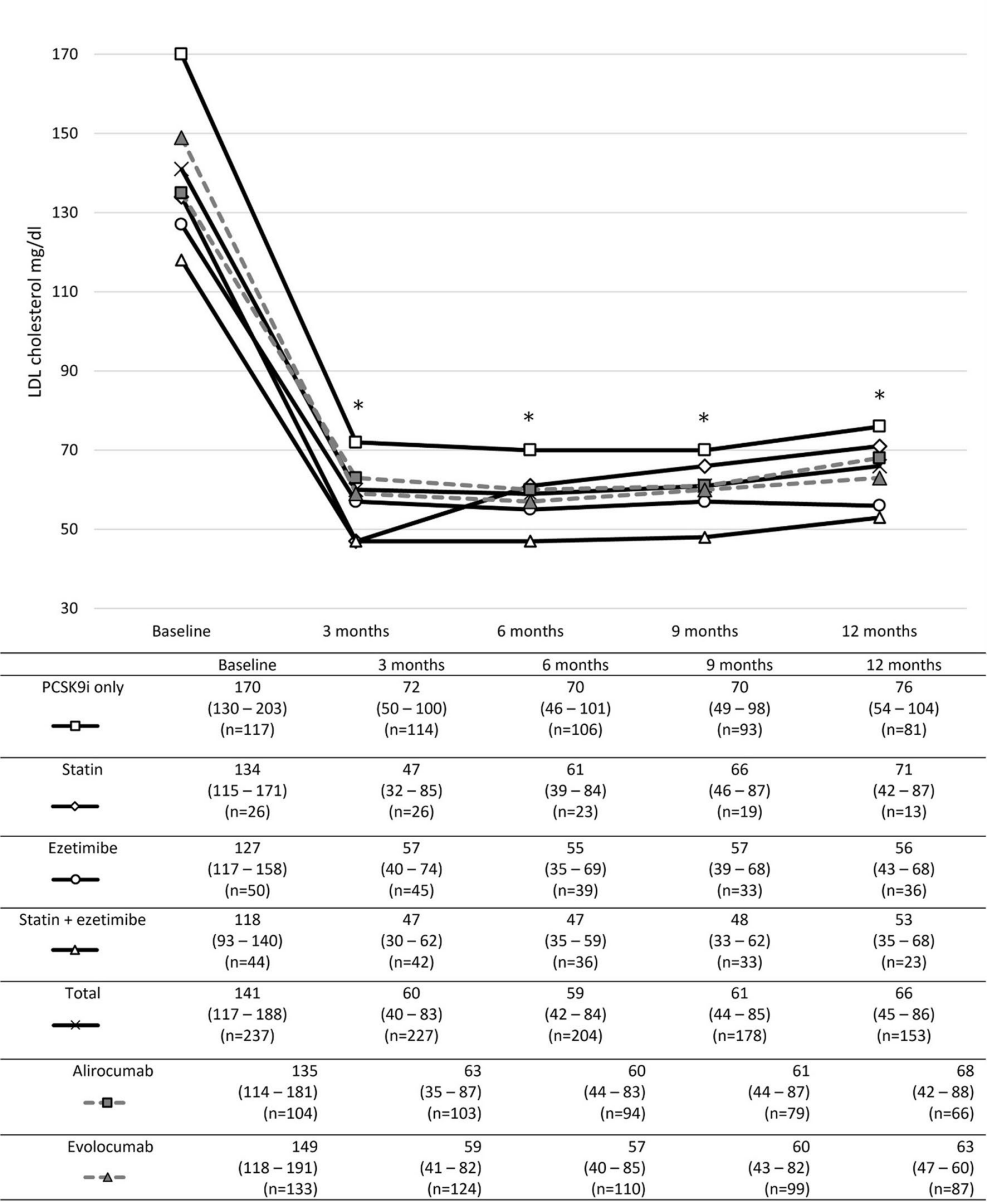
LDL-C levels of patients according to lipid-lowering medication at baseline; all patients were on PCSK9 inhibitor therapy. Data are median (interquartile range). *Indicates statistical significance when compared to baseline LDL-C levels for all groups investigated

LDL-C levels at baseline of patients with T2D were comparable to those without diabetes. At some points of the analysis (at months 6 and 12), a significantly higher reduction of LDL-C was observed in patients with T2D (Fig. 3). Achievement of LDL-C targets < 55 mg/dl after 3 months on therapy was numerically but not significantly higher in patients with T2D compared to those without: < 55 mg/dl: 51% vs. 41.5%; < 70 mg/dl 69.4 vs. 58.5% (p = 0.119). At 12 months, LDL-C treatment targets were achieved in patients with T2D compared to those without < 55 mg/dl: 58.8% vs. 30.1%; < 70 mg/dl 70.6 vs. 49.6% (p = 0.003). Patients with inadequately controlled T2D (HbA1c > 54 mmol/mol) showed a higher but not statistically significant LDL-C reduction at month 12, than T2D patients with baseline HbA1c ≤ 54 mmol/mol (p = 0.052). Detailed data on LDL-C according to diabetes status are shown in Fig. 2 and in Additional file 1: Table S2.

### LDL-C reduction according to adjunct lipid-lowering medication

Considering additive lipid-lowering medication at baseline, a significant reduction in LDL-C was observed in all groups during all observation points which remained stable over time. The greatest decline was seen in patients who were on concomitant combination therapy of a statin and ezetimibe, while the smallest effect on LDL-C reduction was seen in those who did not have adjunct lipid-lowering therapy apart from the PCSK9 inhibitor therapy (Fig. 3). When comparing LDL-C levels at different time points, there was no significant difference in the numerical reduction of LDL-C between these groups (Additional file 1: Table S2).

### Effect on triglyceride levels

Triglyceride (TG) levels slightly decreased from baseline (138 mg/dl) over time: 107 mg/dl (month 3) months, 110 mg/dl (month 6), 111 mg/dl (month 9) and 114 mg/dl (month 12) (p = 0.001). Median numerical and percental changes are shown in Additional file 1: Table S4. There was no significance in the TG reduction between alirocumab and evolocumab. Patients with T2D had significantly higher TG levels at baseline compared to those without diabetes (185 and 124 mg/dl; p = 0.001), TG reduction during treatment was comparable between patients with and without T2D (n.s.). Further details related to TG reduction over time considering PCSK9 inhibitor used and diabetes status is demonstrated in Additional file 1: Table S3.

### Effect on high-density lipoprotein

In the total population, high-density lipoprotein (HDL) slightly increased from baseline (54 mg/dl) at months 3 and 12 (57 mg/dl), respectively. The HDL increase was not significantly sustained after 12 months in the alirocumab treated group. Patients with T2D had significantly lower HDL levels at baseline when compared to patients without T2D. Similar to the overall group, there was a slight increase of HDL in both patients without and with T2D (n.s.). Further details are shown in Additional file 1: Table S4.

### Effect on lipoprotein (a)

Follow-up data on lipoprotein (a) [Lp(a)] was only available in 26 patients, due to infrequent measurements in routine care based on current guideline recommendations (single measurement recommended at least once in a lifetime, not subsequent) [12]. Lp(a) decreased from 67 mg/dl at baseline to 55 mg/dl at the first follow-up measurement (after median 95 days [28–190] from baseline) in these patients. The significant decrease of Lp(a) shown for patients without diabetes, could not be confirmed in patients with T2D (only data from 5 patients available). Further details can be found in Additional file 1: Table S5.

### Effects on HbA1c

The baseline HbA1c of the overall cohort was 41 (37–49) mmol/mol and did not significantly change during the observation period of up to 18 months. Also, in patients with T2D (baseline HbA1c 52 [48–57] mmol/mol) no significant changes of HbA1c were found over time. Detailed data on HbA1c can be found in Additional file 1: Table S6.

Adjustments of the antidiabetic therapy during the study period occurred in 15 patients (T2D, n = 14, T1D, n = 1). Drug treatment intensifications were performed in 9 patients: new or additional oral antidiabetic drug (OAD) or Glucagon-like peptide 1 receptor agonist (GLP1-RA) in 3 patients, increase of OAD dose (n = 1), increase of insulin dose in 4 patients (n = 3 in T2D, n = 1 in T1D), additional OAD plus increase of insulin dose (n = 1). A switch from one class of OAD to another was made in five patients. A reduction of insulin dose occurred in one patient.

### Cardiovascular outcomes

**Fig. 4:**
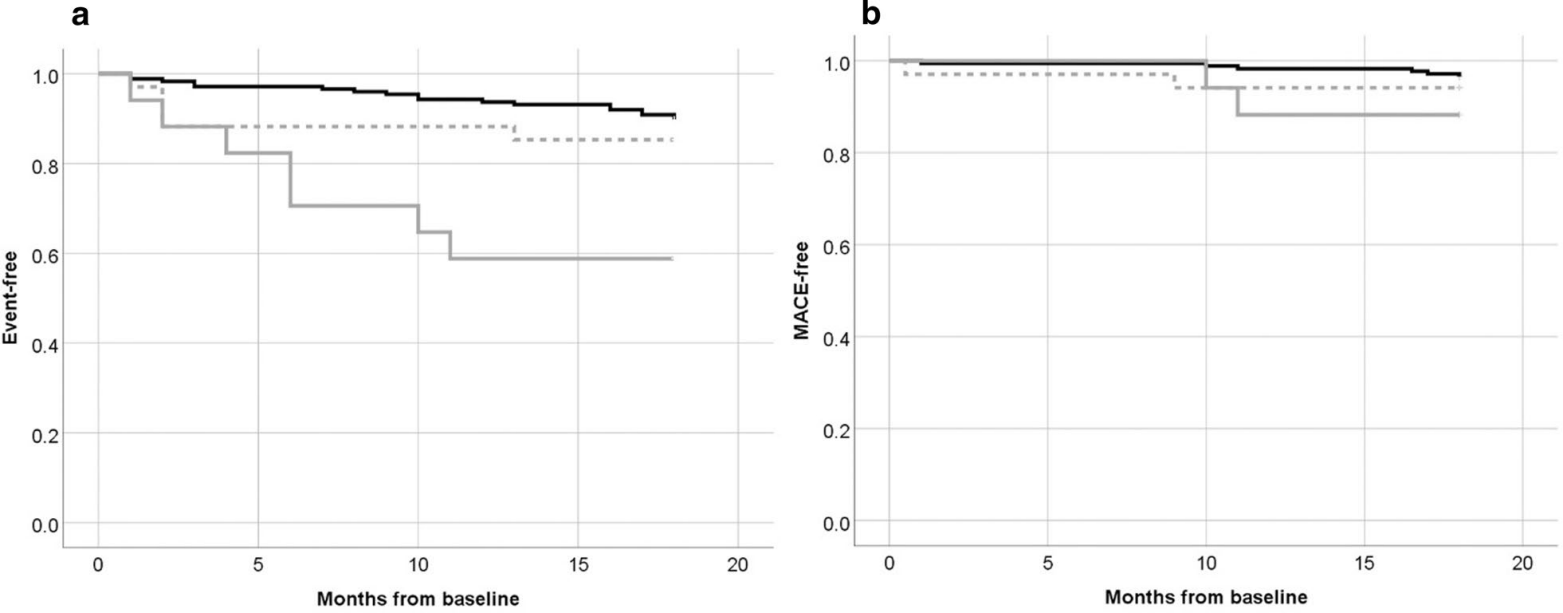
**a** Event-free and **b** MACE-free survival curves of patients without diabetes mellitus (black line), with T2D and baseline HbA1c ≤ 54 mmol/mol (dashed grey line), and with T2D and baseline HbA1c of > 54 mmol/mol (solid grey line). MACE was defined as composite endpoint including non-fatal myocardial infarction (STEMI or NSTEMI), indication of urgent need for coronary intervention, non-fatal stroke or TIA, and cardiovascular death

A total of 42 cardiovascular events in 36 patients were observed during the 18 months observation period; 12 of those were patients with T2D. A composite of major adverse cardiac events (MACE) including non-fatal myocardial infarction (ST—segment elevation myocardial infarction [STEMI] or non-ST—segment elevation myocardial infarction [NSTEMI]), indication of urgent need for coronary intervention, non-fatal stroke or TIA, and cardiovascular death occurred 15 times in 13 patients, four of those with T2D. Patients with insufficiently controlled T2D (HbA1c > 54 mmol/mol) experienced a higher risk of cardiovascular events (HR: 5.1 [2.2–12.4]; p < 0.001) when compared to patients without diabetes mellitus. The total event-rate in those with well controlled T2D (HbA1c ≤ 54 mmol/mol) not was comparable (HR: 1.5 [0.6–4.1; p = 0.410]) to those without diabetes mellitus. The MACE did not significantly differ between those with and without T2D (HR: 2.3 [0.6–7.9]; p = 0.212). Kaplan–Meier survival curves indicating event-free and MACE-free survival are shown in Fig. 4.

### Therapy adjustments, discontinuation, adverse effects

In nine patients the PCSK9 inhibitor agent was altered over time (i.e. change from one agent to the other). Seven patients discontinued PCSK9 inhibitor treatment due to side-effects (mostly because of joint or muscle pain, skin lesions or pruritus, or gastrointestinal symptoms), one because of insufficient response to PCSK9 inhibitor therapy, one did not fulfill the criteria for health insurance coverage of PCSK9 inhibitor treatment (ongoing smoking) and one discontinued by the patient’s own request. One patient with T1D reported on elevated insulin requirements and mild ketoacidosis after 4 months of PCSK9 inhibitor therapy leading to cessation of treatment.

Adverse effects of PCSK9 inhibitor therapy were documented in 27 patients. The majority of side effects was reported from patients without concomitant lipid lowering therapy (20 patients; 74%); all of the reported effects were of mild quality. The most frequent side effects were joint or muscle pain, rhinitis, flu-like symptoms, fatigue, skin lesions or pruritus. Side effects associated with the PCSK9 inhibitor therapy and the motivations for therapy adjustments are shown in Table 3.

**Table 3:** Side effects of PCSK9 inhibitor therapy reported by patients, cause of therapy adjustments and discontinuation

Adverse effect documented in patients record	
Joint or muscle pain	10
Rhinitis	5
Flu-like symptoms	5
Fatigue	4
Skin lesions or pruritus	4
Gastrointestinal symptoms	2
Insomnia	1
Elevated insulin requirements	1
Cause leading to PCSK9 inhibitor therapy adjustment (switch of PCSK9 inhibitor agent)	
Adverse effect	8
Joint or muscle pain	6
Flu-like symptoms	3
Fatigue	1
Gastrointestinal symptoms	1
Dose reduction (evolocumab 140 mg to alirocumab 75 mg)	1
Cause leading to PCSK9 inhibitor therapy discontinuation	
Adverse effect	7
Joint or muscle pain	3
Skin lesions or pruritus	2
Gastrointestinal symptoms	1
Adverse effects not specified	1
Elevated insulin requirement	1
Non-responder (clearly documented)	1
Reasons of insurance coverage	1
By patient’s request	1

Data are number of patients. Patients could have more than one symptom

## Discussion

This real-world data analysis evaluated data from patients in a tertiary center who received PCSK9 inhibitors for the treatment of hypercholesterolemia. Within this setting PCSK9 inhibitors were mainly prescribed in secondary prevention for patients with established cardiovascular disease and multiple cardiovascular risk factors. Statin intolerance and side-effects of ezetimibe was reported in the majority of cases (83.1% and 44.7, respectively) and appears to be a main driver for prescription of PCSK9 inhibitor therapy. This frequent intolerance to these first-line agents explains why about half of the patients had no lipid-lowering medication on board at the time of the first prescription of a PCSK9 inhibitor.

In our investigation, the significant reduction of LDL-C levels which was comparable with both agents after only a few weeks underlines the effective potential of PCSK9 inhibitor drugs as lipid-lowering therapy. RCTs like the FOURIER and ODYSSEY OUTCOMES trials showed impressive results in lowering LDL-C. As compared to our study, in the FOURIER trial, substantially more patients achieved LDL-C levels < 70 mg/dl and < 40 mg/dl (87% and 67% vs. 56.2% and 19.6% in our study). In both RCTs a reduction to even lower LDL-C levels was achieved (LDL-C of 30 mg/dl after 48 weeks in the FOURIER trial; LDL-C of 48 mg/dl after 12 months in the ODYSSEY OUTCOMES trial vs. LDL-C of 66 mg/dl after 12 months in our study). However, it should be considered that the baseline LDL-C was considerably lower in these RCTs, and that a majority of patients were on concomitant statin therapy (FOURIER: 69.3%; ODYSSEY: 88.8%) with or without ezetimibe (FOURIER: 5.2%; ODYSSEY: 2.9%), which may explain the differences to our data [5, 6]. In routine care, and also as shown by our analysis, there is a high number of patients on PCSK9 inhibitor therapy without concomitant lipid-lowering therapy. In many cases this is due to intolerance against the first-line therapy. This condition thus hinders further intensification of lipid-lowering therapy aside from PCSK9 inhibitor therapy. Within clinical studies, nutrition and adherence to prescribed medication are intensely monitored; this might positively impact LDL-C reductions seen in RCTs and might result in a lesser effect LDL-C reduction by PCSK9 inhibitors in everyday use.

Of note, our population differed from the patients in the ODYSSEY OUTCOMES study, as in this study only patients with recent cardiovascular events (< 12 months) were included [6]. History of cardiovascular diseases was frequent in our patients, but a recent or previous cardiovascular event was not a mandatory criterion to be eligible for analysis.

Our study showed a similar percentage of LDL-C reduction reported in a systematic review on PCSK9 inhibitors (LDL-C reduction of 53.86% at month 6) [13]. Real-world studies also observed inter-individual reduction of LDL-C and postulated that LDL-C goals cannot be reached in all patients [14–16]. This should be considered in the PCSK9 inhibitor treatment in clinical practice. Achievement of treatment goals should be regularly monitored in routine care with special attention to treatment adherence and therapy intensification, if possible.

In a prespecified analysis of the ODYSSEY OUTCOMES study comparing the effect of alirocumab on patients with diabetes, prediabetes and normoglycemia, no significant differences in LDL-C reduction were found across these subgroups (median LDL-C reduction of 64–65% at month 4) [17]. This finding was confirmed in another sub-cohort of the ODYSSEY collective [18]. In a sub-study of the FOURIER study, no differences in LDL-C reduction were seen when evolocumab was used in patients with and without diabetes (− 57% in patients with and − 60% in patients without diabetes) [7]. In our study, PCSK9 inhibitor therapy markedly lowered LDL-C in both patients with and without diabetes irrespective of glycemic control. Both, the FOURIER and the ODYSSEY OUTCOMES trial found a more pronounced absolute risk reduction of a composite of major cardiovascular events in patients with diabetes mellitus receiving PCSK9 inhibitors compared to those without diabetes [7, 17]. Patients with T2D are at an extraordinary increased cardiovascular risk. Therefore, patient with T2D and hypercholesterinemia especially benefit in particular from an intensified lipid-lowering treatment.

Clinical trials and genetic studies have ascertained a potential link between the use of lipid-lowering therapy and an increased risk for deterioration of glucose control and the development of T2D. In statin users the risk to develop T2D was estimated to be approximately 1:1.000 per year of exposure and patients with additional risk factors to develop T2D (e.g. age, prediabetes, metabolic syndrome) were at an even increased risk [19]. A Chinese study reported on the association between elevated circulating PCSK9 levels and an increased risk for T2D in women with prediabetes [20].

Schmidt et al. investigated the relation of PCSK9 gene variants and glycemic parameters in a mendelian study, suggesting a potential risk for new-onset diabetes in patients on PCSK9 inhibitor treatment. Mimicking the pharmacological effects of PCSK9 inhibition, this study found increased glucose concentrations and an increased risk of T2D in carriers of PCSK9 variants associated with low LDL-C levels. However, these findings represent the life-long effect of these gene variants and may not reflect the pharmacological PCSK9-targeted intervention later in life [10].

No significant changes on patients’ Hb1Ac levels were found in our study, in both patients with and without preexisting diabetes. A limitation of our study was that only in a small number of patients all investigated parameters were available at all time-points. Nevertheless, the results are consistent with findings from past studies, which showed no relevant changes in glycemic control during PCSK9 inhibitor therapy [8, 21–24].

In our study we observed adjustments and intensifications of diabetes therapy when required. Of note, due to refund claims by the Austrian health insurance only patients with sufficiently controlled diabetes (HbA1c < 64 mmol/mol (8.0%)) are eligible for PCSK9 inhibitor therapy. Therefore, this study cannot provide data on potential effects of PCSK9 inhibitor therapy on glucose control, LDL-C and other parameters of the lipid panel as well as cardiovascular outcome in patients with insufficiently controlled diabetes mellitus.

While there is a plenty of observational data available demonstrating a reduction of cardiovascular outcomes by therapeutically reduce LDL-C [25, 26], to the best of our knowledge there is no real-world evidence available investigating these potentially beneficial effects when populations with PCSK9 inhibitor therapy were selected.

It has been shown that low PCSK9 levels are associated with the presence of metabolic syndrome and atherosclerosis in patients with coronary heart disease [27] and recently, a study conducted by Peng et al. described an important relationship between elevated circulating PCSK9 levels to be associated with higher cardiovascular morbidity and adverse cardiovascular outcomes specifically in patients with diabetes [28]. The authors suggest to measure PCSK9 levels in patients with diabetes to identify the ones with particularly high cardiovascular risk, indicating that these patients might be the ones who might even profit from lower LDL-targets (e.g. < 40 mg/dl) established by therapeutic PCSK9 inhibition [28, 29].

Within our population, 42 cardiovascular events of any kind during an 18 months observation, of those 15 events met the criteria to be characterized as a major adverse cardiovascular outcome event (MACE). Noteworthy, we have found significantly more all cause events in patients with T2D at baseline and intriguingly, despite a higher LDL reduction than the non-diabetic population those patients with T2D with HbA1c > 54 mmol/mol experienced a fivefold higher risk of any cardiovascular event compared to those without diabetes. This finding emphasizes diabetes to be a further potent cardiovascular risk factor, specifically when insufficiently controlled which needs to be accordingly paid attention.

In our analysis we observed a reduction in triglyceride levels of about 20% throughout the observational period. This finding has to be interpreted with caution as patients do not mandatorily need to be in a fasting state for blood sampling in our clinic. Still, this result is in-line with previous research indicating a 12–30% reduction of triglycerides due to PCSK9 inhibitor therapy [30, 31]. In our population, this finding might be attributed to various factors: first, due to the national reimbursement criteria, all patients must receive professional nutritional advice prior to initiation of PCSK9 inhibitor therapy. Secondly, previous data suggests that intra- and extracellular PCSK9 might act in a complementary fashion to regulate triglyceride levels, on the one hand, intracellularly, by the ability of PCSK9 to modulate apolipoprotein B (APOB) secretion and on the other hand, extracellularly, by triggering LDL-receptor mediated catabolism [32]. Further investigations, focusing on the physiologic mechanisms which might explain the positive correlation of PCSK9 inhibitor therapy on triglyceride levels are in progress.

A further well-known cardiovascular risk factor is lipoprotein(a) [Lp(a)]. At present there are no approved medical agents to reduce Lp(a) available. Lp(a) currently can be lowered only by means of lipid apheresis and to some extent by dietary interventions [12]. In our study Lp(a) data over time are only available to a very limited extent. This is caused by current guidelines that suggest only a single Lp(a) determination to determine whether lipid apheresis might be necessary [12]. In secondary analyses of the FOURIER and the ODYSSEY OUTCOMES trials, alirocumab and evolocumab demonstrated only a small reduction (− 20%) of Lp(a) [33, 34], which is similar to the results seen in our study. A novel and not yet approved medical approach (antisense-oligonucleotide therapy) might lead to clinically relevant Lp(a) reduction in the future [35].

In our analysis, adverse effects of alirocumab or evolocumab were rare. Since the documented symptoms stated by the patients were mainly unspecific (e.g. joint or muscle pain, rhinitis), it remains speculative whether they were associated with PCSK9 inhibitor therapy. Most of the adverse events leading to discontinuation of PCSK9 inhibitor therapy occurred in patients without concomitant lipid-lowering therapy. Thus, the potential side effect can probably truly be attributed to the PCSK9 inhibitor therapy. The adverse effects described in our study are similar to those found in other studies [5, 6, 36, 37]. Overall, PCSK9 inhibitors appear were tolerated, although long-term data on adverse health effects are not available yet.

The strength of this retrospective study is the large sample size of 237 patients. Many real-world studies available have investigated only one of the two agents and were mainly sponsor-initiated studies [38]. Furthermore, real-world evidence is substantially lacking investigating the efficacy of PCSK9 inhibitor therapy on LDL-C reduction in patients with diabetes. In our study we describe for the first time, that patients with insufficiently controlled T2D might have a superior LDL-C reduction by PCSK9 inhibitor therapy compared to patients without diabetes mellitus. This finding needs to be addressed in prospective randomized controlled clinical trials. Additionally, special attention should be given to patients with. Also, insufficiently controlled T2D as glycemic control seems to have a relevant strong impact on cardiovascular events despite achieving LDL-C treatment goals. This finding might be attributed to the fact that in our population the proportion of patients on sodium glucose co-transporters 2 (SGLT2) inhibitors [39–41] and GLP1-RA [42, 43] was rather low at baseline. Both agents have demonstrated beneficial cardiovascular outcome in patients with T2D. The broad uptake of these medications into routine care only occurred in more recent years following guideline recommendations [44]. In the beginning these agents were mainly prescribed by diabetologists and endocrinologists whereas a large proportion of our patients were seen by a GP for diabetes care until the first presentation at our center. Potential beneficial cardiovascular effects might be expected in patients with diabetes receiving both PCSK9 inhibitor therapy as lipid-lowering agent and SGLT2 inhibitors and/or GLP1-RA for diabetes management.

By the retrospective nature of the study several limitations have to be addressed. The major limitation is the varying availability of data, especially laboratory data. This is due to the fact that in routine clinical care, no stringent protocol as in a clinical trial is applied. Many of our patients performed the laboratory analysis with their GP who might only chose a limited laboratory panel than recommended in our patient letters. Additionally, follow-up visits in many cases are performed via telemedical methods (i.e. video call, telephone call) which has a negative impact on availability of laboratory measurements or further baseline characteristics as BMI or blood pressure.

There was a relatively large number of patients (11.4%; n = 27) who were lost to follow-up (LTF). This might result in some selection bias and thus can impact the results. One can assume that more compliant and adherent patients thus are represented by our data. Also, no telephone follow-up was performed due to the nature of the study. So, there might even have been a fatal event in one of patients LTF, as only fatalities within the Styrian hospital cluster (KAGES) and adjacent hospitals would have been noticed.

Additionally, concurrent lipid-lowering medication was ascertained only at baseline and potential adjustments were not considered in the analysis. Further, adherence to lifestyle recommendations were not systematically documented. As all patients who are prescribed with and reimbursed for PCSK9 inhibitor therapy obtain professional nutritional advice, the effect of this training should be evenly distributed among the investigated population.

Another limitation is the lack of continuous documentation of further cardiovascular risk factors such as body weight, body mass index or blood pressure. This is due to the fact that the enrolled patients presented in a specialized clinic for lipid metabolism disorders which focuses their recommendations on metabolic disease (lipids and diabetes); other cardiovascular risk factors are adjusted elsewhere. Are not taken and the patient is not always available on-site.

## Conclusions

In conclusion, significant reductions in LDL-C and a high percentage of patients achieving the recommended treatment targets were observed in a real-world population over the course of 18 months. Interestingly, patients with T2D, and specifically those with insufficiently controlled T2D showed a superior response to PCSK9 inhibitor therapy. Still, they experienced a substantial number of cardiovascular events. Thus, efforts also must be made to achieve better glycemic control. Even when receiving PCSK9 inhibitor therapy, currently the most potent lipid-lowering agent, some patients did not meet LDL-C treatment goals as recommended in current guidelines. Special attention to these patients is required in the future to achieve these goals to avoid subsequent adverse cardiovascular outcomes.

## Supplementary Information

**Additional file 1:** **Table S1**. Cardiovascular disease at baseline (by medical condition). Data are number of patients (% from subgroup). **Table S2.** LDL-C levels over time of the overall cohort and by PCSK9 inhibitor agent (A) and according to diabetes status (B). Data are median (IQR). *P-values were calculated by using the Wilcoxon signed-rank test and refers to the comparisons of laboratory data between baseline and defined time-point. **Table S3.** Triglyceride levels over time of the overall cohort and by PCSK9 inhibitor (A) and according to diabetes status (B). Data are median (IQR). *P-values were calculated by using the Wilcoxon signed-rank test and refers to the comparisons of laboratory data between baseline and defined time-point. **Table S4.** HDL cholesterol levels over time of the overall cohort and by PCSK9 inhibitor agent (A) and diabetes status (B). Data are median (IQR). *P-values were calculated by using the Wilcoxon signed-rank test and refers to the comparisons of laboratory data between baseline and defined time-point. **Table S5.** Lipoprotein(a) levels and follow-up measurement of the overall cohort, by PCSK9 inhibitor agent and by diabetes status. Data are median (IQR). *P-values were calculated by using the Wilcoxon signed-rank test and refers to the comparisons of laboratory data between baseline and defined time. Only patients with follow-up data for Lp(a) were included in the analysis. † First performed follow-up measurement after initiation of PCSK9 inhibitor therapy was included in the analysis. **Table S6.** HbA1c levels over time of the overall cohort, without and with diabetes mellitus at baseline. Data are median (IQR). * P-values were calculated by using the Wilcoxon signed-rank test and refers to the comparisons of laboratory data between baseline and defined time-point.

## Data Availability

Data was extracted from medical records and saved on a separate password-protected file with anonymized data.

## Abbreviations

ASCVD: Atherosclerotic cardiovascular disease
EAS: European Atherosclerosis Society
ESC: European Society of Cardiology
FH: Familial hypercholesterolemia
GLP1: Glucagon- like peptide 1
HbA1c: Hemoglobin A1c
HDL-C: High-density lipoprotein cholesterol
LDL-C: Low-density lipoprotein cholesterol
Lp(a): Lipoprotein (a)
LTF: Lost to follow up
PCSK9: Proprotein convertase subtilisin/kexin type 9
RCTs: Randomized controlled trials
SGLT2: Sodium-glucose co-transporter 2
T1D: Diabetes mellitus type 1
T2D: Diabetes mellitus type 2
TIA: Transient ischemic attack
TG: Triglycerides
