# Supplementary material for: Real-world data on metabolic effects of PCSK9 inhibitors in a tertiary care center in patients with and without diabetes mellitus
Source: Cardiovasc Diabetol. 2021 Apr 24;20:89. doi: 10.1186/s12933-021-01283-w (PMC8070307; doi:10.1186/s12933-021-01283-w)
Supplement: Supplementary file 1 — Additional file 1: Table S1. Cardiovascular disease at baseline (by medical condition). Data are number of patients (% from subgroup). Table S2. LDL-C levels over time of the overall cohort and by PCSK9 inhibitor agent (A) and according to diabetes status (B). Data are median (IQR). *P-values were calculated by using the Wilcoxon signed-rank test and refers to the comparisons of laboratory data between baseline and defined time-point. Table S3. Triglyceride levels over time of the overall cohort and by PCSK9 inhibitor (A) and according to diabetes status (B). Data are median (IQR). *P-values were calculated by using the Wilcoxon signed-rank test and refers to the comparisons of laboratory data between baseline and defined time-point. Table S4. HDL cholesterol levels over time of the overall cohort and by PCSK9 inhibitor agent (A) and diabetes status (B). Data are median (IQR). *P-values were calculated by using the Wilcoxon signed-rank test and refers to the comparisons of laboratory data between baseline and defined time-point. Table S5. Lipoprotein(a) levels and follow-up measurement of the overall cohort, by PCSK9 inhibitor agent and by diabetes status. Data are median (IQR). *P-values were calculated by using the Wilcoxon signed-rank test and refers to the comparisons of laboratory data between baseline and defined time. Only patients with follow-up data for Lp(a) were included in the analysis. † First performed follow-up measurement after initiation of PCSK9 inhibitor therapy was included in the analysis. Table S6. HbA1c levels over time of the overall cohort, without and with diabetes mellitus at baseline. Data are median (IQR). * P-values were calculated by using the Wilcoxon signed-rank test and refers to the comparisons of laboratory data between baseline and defined time-point. [file 12933_2021_1283_MOESM1_ESM.docx]

Additional Appendix

|  | Coronary heart disease | Stroke/TIA | Carotid artery disease | Peripheral artery disease | Retinopathy | Arterial hypertension | Chronic kidney disease | Age ≥65 years | Diabetes mellitus | Smoking |
| --- | --- | --- | --- | --- | --- | --- | --- | --- | --- | --- |
| Coronary heart disease  (n=177) | - | 17  9.6% | 40  22.6% | 21  11.9% | 8  4.5% | 132  74.6% | 18  10.2% | 95  53.7% | 43  24.3% | 7  4.0% |
| Stroke or TIA  (n=32) | 17  53.1% | - | 14  43.8% | 6  18.8% | 0  0% | 23  71.9% | 5  15.6% | 19  59.4% | 8  25.0% | 0  0% |
| Carotid artery disease  (n=73) | 40  54.8% | 14  19.2% | - | 19  26.0% | 2  2.7% | 51  69.9% | 6  8.2% | 49  67.1% | 22  30.1% | 4  5.5% |
| Peripheral artery disease  (n=43) | 21  48.8% | 6  14.0% | 19  44.2% | - | 3  7.0% | 33  76.7% | 7  16.3% | 23  53.5% | 18  41.9% | 7  16.3% |
| Retinopathy  (n=10) | 8  80.0% | 0  0% | 2  20.0% | 3  30.0% | - | 7  70.0% | 3  30.0% | 5  50.0% | 6  60.0% | 1  10.0% |
| Arterial hypertension  (n=163) | 132  81.0% | 23  14.1% | 51  31.3% | 33  20.2% | 7  4.3% | - | 19  11.7% | 94  57.7% | 51  31.3% | 8  4.9% |
| Chronic kidney disease  (n=23) | 18  78.3% | 5  21.7% | 6  26.1% | 7  30.4% | 3  13.0% | 19  82.6% | - | 18  78.3% | 13  56.5% | 0  0% |
| Age ≥65 years  (n=123) | 95 77.2% | 19 15.4% | 49  39.8% | 23  18.7% | 5 4.1% | 94  76.4% | 18  14.6% | - | 36  29.3% | 3  2.4% |
| Diabetes mellitus  (n=62) | 43  69.4% | 8  12.9% | 22  35.5% | 18  29.0% | 6  9.7% | 51  82.3% | 13  21.0% | 36  58.1% | - | 2  3.2% |
| Current smoker  (n=14) | 7  50.0% | 0  0% | 4  28.6% | 7  50.0% | 1  7.1% | 8  57.1% | 0  0% | 3  21.4% | 2  14.3% | - |

**Table S1**. Cardiovascular disease at baseline (by medical condition). Data are number of patients (% from subgroup).

|  | **Overall cohort** | | **Alirocumab** | | | | **Evolocumab** | | |
| --- | --- | --- | --- | --- | --- | --- | --- | --- | --- |
| **A** | LDL cholesterol  (mg/dl) | p-value * | % LDL-C reduction | LDL cholesterol  (mg/dl) | p-value * | % LDL-C reduction | LDL cholesterol  (mg/dl) | p-value * | % LDL-C reduction |
| **Baseline** | 141  (117 – 188)  (n=237) |  | n.a. | 135  (114 – 181)  (n=104) |  | n.a. | 149  (118 – 191)  (n=133) |  | n.a. |
| **Month 3** | 60  (40 – 83)  (n=227) | <0.001 | 57.5 (42.4 – 70.5) | 63  (35 – 87)  (n=103) | <0.001 | 55.3  (40.4 – 71.3) | 59  (41 – 82)  (n=124) | <0.001 | 60.8  (45.0 – 70.3) |
| **Month 6** | 59 (42 – 84)  (n=204) | <0.001 | 57.0 (45.8 – 70.1) | 60  (44 – 83)  (n=94) | <0.001 | 54.5  (45.7 – 68.7) | 57  (40 – 85)  (n=110) | <0.001 | 60.8  (45.8 – 70.6) |
| **Month 9** | 61 (44 – 85)  (n=178) | <0.001 | 57.1 (45.5 - 68.7) | 61  (44 – 87)  (n=79) | <0.001 | 54.2  (41.9 – 68.3) | 60  (43 – 82)  (n=99) | <0.001 | 59.5  (46.6 – 70.3) |
| **Month 12** | 66 (45 – 86)  (n=153) | <0.001 | 53.6  (42.5 - 66.7) | 68  (42 – 88)  (n=66) | <0.001 | 52.5  (40.7 – 67.0) | 63  (47 – 60)  (n=87) | <0.001 | 53.6  (43.8 – 66.4) |

| **B** | **Without diabetes mellitus** | **T2D** | **p-value *** | **T2D HbA1c**  **≤54** | **T2D HbA1c >54** | **p-value *** |
| --- | --- | --- | --- | --- | --- | --- |
| Baseline | (n=175) | (n=54) |  | (n=34) | (n=17) |  |
| LDL-C (mg/dl) | 141  (117 – 188) | 135  (110 – 178) | 0.327 | 130  (104 – 174) | 148  (119 – 192) | 0.250 |
| Month 3 | (n=171) | (n=49) |  | (n=32) | (n=14) |  |
| LDL-C (mg/dl) | 62  (44 – 83) | 55  (34 – 80) | 0.176 | 58  (35 – 86) | 41  (30 – 63) | 0.197 |
| % LDL-C reduction | 55.9  (41.3 – 69.4) | 62.3  (46.4 – 72.0) | 0.239 | 61.1  (43.0 – 69.4) | 70.5  (61.9 – 81.6) | 0.025 |
| Month 6 | (n=154) | (n=45) |  | (n=29) | (n=13) |  |
| LDL-C (mg/dl) | 61  (44 – 85) | 48  (36 – 73) | 0.034 | 48  (37 – 77) | 41  (30 – 63) | 0.615 |
| % LDL-C reduction | 55.4  (44.4 – 68.8) | 64.4  (53.9 – 72.0) | 0.012 | 63.8  (54.5 – 68.4) | 70.5  (57.7 – 81.3) | 0.100 |
| Month 9 | (n=132) | (n=40) |  | (n=26) | (n=11) |  |
| LDL-C (mg/dl) | 62  (44 – 87) | 53  (39 – 84) | 0.215 | 52  (39 – 83) | 51  (34 – 92) | 0.740 |
| % LDL-C reduction | 55.5  (43.9 – 67.8) | 62.6  (48.9 – 71.1) | 0.157 | 62.6  (47.7 – 70.8) | 66.4  (54.5 – 75.3) | 0.319 |
| Month 12 | (n=113) | (n=34) |  | (n=22) | (n=9) |  |
| LDL-C (mg/dl) | 71  (49 – 91) | 52  (39 – 78) | 0.007 | 58  (40 – 82) | 40  (30 – 55) | 0.041 |
| % LDL-C reduction | 50.8  (41.3 – 63.6) | 64.5  (47.4 – 73.3) | 0.010 | 57.7  (38.2 – 71.1) | 66.8  (63.6 – 86.4) | 0.041 |

**Table S2.** LDL-C levels over time of the overall cohort and by PCSK9 inhibitor agent (A) and according to diabetes status (B). Data are median (IQR). * P-values were calculated by using the Wilcoxon signed-rank test and refers to the comparisons of laboratory data between baseline and defined time-point.

| **A** | **Overall cohort** | | | **Alirocumab** | | | **Evolocumab** | | |
| --- | --- | --- | --- | --- | --- | --- | --- | --- | --- |
|  | Triglycerides  (mg/dl) | p-value * | % change from baseline | Triglycerides  (mg/dl) | p-value * | % change from baseline | Triglycerides  (mg/dl) | p-value * | % change from baseline |
| **Baseline** | 138  (99 – 215)  (N=227) |  | n.a. | 138  (102 – 255)  (n=102) |  | n.a. | 138  (97 – 197)  (n=125) |  | n.a. |
| **Month 3** | 107  (77 – 170)  (n=218) | <0.001 | -19.5  (-36.9 – +1.0) | 109  (86 – 169)  (n=101) | <0.001 | -19.4  (-35.8 – +5.1) | 106  (73 – 173)  (n=117) | <0.001 | -20.0  (-37.9 – 0.0) |
| **Month 6** | 110  (74 – 181)  (n=195) | <0.001 | -19.5  (-37.5 – +0.7) | 116  (85 – 202)  (n=92) | <0.001 | -17.7  (-32.3 – 0.0) | 106  (70 – 173)  (n=103) | <0.001 | -22.1  (-41.5 – +2.5) |
| **Month 9** | 111  (80 – 169)  (n=152) | 0.004 | -16.9  (-38.5 – +3.1) | 113  (82 – 189)  (n=64) | <0.001 | -13.1  (-34.0 – +0.5) | 110  (76 – 154)  (n=88) | <0.001 | -22.6  (-40.2 – +3.8) |
| **Month 12** | 114  (84 - 176)  (n=118) | 0.001 | -10.4  (-30.8 – +10.3) | 121  (73 – 201)  (n=41) | 0.014 | -13.1  (-23.0 – +7.8) | 111  (85 – 169)  (n=77) | 0.019 | -8.2  (-31.3 – +11.4) |
| **B** | **Without diabetes mellitus** | | | **T2D** | | | **p-value*** | | |
| **Baseline** | (n=167) | | | (n=52) | | |  | | |
| **Triglycerides** (mg/dl) | 124  (97 – 199) | | | 185  (134 – 249) | | | 0.001 | | |
| **Month 3** | (n=164) | | | (n=47) | | |  | | |
| **Triglycerides** mg/dl | 100  (72 – 160) | | | 161  (106 – 231) | | | >0.001 | | |
| **% Triglyceride change** | -19.7  (-37.0 – -0.8) | | | -15.3  (-35.3 – +9.8) | | | 0.205 | | |
| **Month 6** | (n=146) | | | (n=44) | | |  | | |
| **Triglycerides**  (mg/dl) | 101  (69 – 161) | | | 171  (99 – 243) | | | >0.001 | | |
| **% Triglyceride change** | -22.9  (-39.2 – -1.9) | | | -11.2  (-30.6 – +5.8) | | | 0.073 | | |
| **Month 9** | **(n=111)** | | | **(n=36)** | | |  | | |
| **Triglycerides** (mg/dl) | 101  (73 – 154) | | | 152  (100 – 216) | | | 0.001 | | |
| **% Triglyceride change** | -17.8  (-38.6 – 0.0) | | | -11.0  (-31.9 – +14.9) | | | 0.140 | | |
| **Month 12** | (n=86) | | | (n=29) | | |  | | |
| **Triglycerides**  (mg/dl) | 101  (77 – 142) | | | 177  (100 – 231) | | | >0.001 | | |
| **% Triglyceride change** | -13.7  (-31.3 – +5.2) | | | -1.1  (-25.7 – +27.4) | | | 0.084 | | |

**Table S3.** Triglyceride levels over time of the overall cohort and by PCSK9 inhibitor (A) and according to diabetes status (B). Data are median (IQR). * P-values were calculated by using the Wilcoxon signed-rank test and refers to the comparisons of laboratory data between baseline and defined time-point.

| **A** | **Overall cohort** | | | **Alirocumab** | | | | **Evolocumab** | | | | |
| --- | --- | --- | --- | --- | --- | --- | --- | --- | --- | --- | --- | --- |
|  | HDL  cholesterol  (mg/dl) | p-value * | % change from baseline | HDL  cholesterol  (mg/dl) | | p-value * | % change from baseline | HDL  cholesterol  (mg/dl) | | p-value * | % change from baseline | |
| **Baseline** | 54  (45 – 65)  (n=226) |  | n.a. | 52  (44 – 63)  (n=100) | |  | n.a. | 55  (46 – 67)  (n=126) | |  | n.a. | |
| **Month 3** | 57  (48 – 68)  (n=217) | <0.001 | +6.5  (-4.0 –+16.5) | 56  (46 – 67)  (n=98) | | 0.007 | +6.1  (-5.0 – +15.2) | 59  (50 – 71)  (n=119) | | <0.001 | +6.7  (-2.5 – +18.5) |  |
| **Month 6** | 57  (47 – 69)  (n=191) | <0.001 | +8.9  (-3.5 – +16.3) | 55  (45 – 66)  (n=87) | | <0.001 | +9.8  (-3.2 – +16.0) | 60  (48 – 71)  (n=104) | | <0.001 | +8.5  (-4.1 – +17.9) |  |
| **Month 9** | 58  (48 – 70)  (n=154) | <0.001 | +8.5  (-3.7 – +18.4) | 56  (49 – 68)  (n=66) | | <0.001 | +9.8  (-3.0 – +18.0) | 59  (47 – 71)  (n=88) | | 0.001 | +8.0  (-4.1 – +20.0) |  |
| **Month 12** | 57  (49 – 69)  (n=121) | 0.010 | +3.8  (-4.6 – +16.7) | 59  (51 – 68)  (n=45) | | 0.156 | +3.4  (-4.5 – +14.2) | 55  (48 – 71)  (n=76) | | 0.028 | +4.3  (-4.8 – +18.0) |  |
| **B** | | **Without diabetes mellitus** | | | **T2D** | | | | **p-value*** | | |  |
| **Baseline** | | (n=169) | | | (n=49) | | | |  | | |  |
| **HDL-C** (mg/dl) | | 54  (46 – 67) | | | 49  (42 – 60) | | | | 0.027 | | |  |
| **Month 3** | | (n=166) | | | (n=44) | | | |  | | |  |
| **HDL-C** (mg/dl) | | 59  (49 – 71) | | | 52  (46 – 62) | | | | 0.008 | | |  |
| **% HDL change** | | +7.3  (-3.6 – +16.2) | | | +4.2  (-6.9 – +19.1) | | | | 0.365 | | |  |
| **Month 6** | | (n=146) | | | (n=40) | | | |  | | |  |
| **HDL-C** (mg/dl) | | 58  (47 – 70) | | | 52  (47 – 67) | | | | 0.232 | | |  |
| **% HDL change** | | +9.1  (-4.0 – +16.0) | | | +7.5  (-1.3 – +20.4) | | | | 0.600 | | |  |
| **Month 9** | | (n=115) | | | (n=35) | | | |  | | |  |
| **HDL-C** (mg/dl) | | 58  (48 – 71) | | | 52  (43 – 68) | | | | 0.192 | | |  |
| **% HDL change** | | +9.5  (-4.3 – +18.4) | | | +6.5  (-2.3 – +20.5) | | | | 0.862 | | |  |
| **Month 12** | | (n=91) | | | (n=28) | | | |  | | |  |
| **HDL-C** (mg/dl) | | 58  (50 – 69) | | | 53  (43 – 69) | | | | 0.200 | | |  |
| **% HDL change** | | +2.9  (-5.0 – +15.1) | | | +5.5  (-1.3 – +17.9) | | | | 0.313 | | |  |

**Table S4.** HDL cholesterol levels over time of the overall cohort and by PCSK9 inhibitor agent (A) and diabetes status (B). Data are median (IQR). * P-values were calculated by using the Wilcoxon signed-rank test and refers to the comparisons of laboratory data between baseline and defined time-point.

|  | **Lp(a)**  **at baseline**  **(mg/dl)** | **Lp(a)**  **follow-up †**  **(mg/dl)** | **p-value*** | **% change from baseline** |
| --- | --- | --- | --- | --- |
| **Overall cohort**  **(n=26)** | 67  (37 – 127) | 55  (34 – 96) | <0.001 | -14.7  (-44.8 – -2.6) |
| **Alirocumab**  **(n=7)** | 105  (90 – 163) | 81  (69 - 110 | 0.028 | -28.2  (-43.6 – -3.5) |
| **Evolocumab**  **(n=19)** | 56  (25 – 80) | 51  (17 – 92) | 0.004 | -14.4  (-48.5 – +0.2) |
| **Without diabetes mellitus**  **(n=21)** | 67  (43 – 151) | 63  (43 – 115) | 0.001 | -15.0  (-38.0 – -4.9) |
| **Diabetes mellitus type 2**  **(n=5)** | 51  (26 – 91) | 42  (13 – 66) | 0.225 | -13.9  (-65.3 – +9.0) |

**Table S5.** Lipoprotein(a) levels and follow-up measurement of the overall cohort, by PCSK9 inhibitor agent and by diabetes status. Data are median (IQR). *P-values were calculated by using the Wilcoxon signed-rank test and refers to the comparisons of laboratory data between baseline and defined time. Only patients with follow-up data for Lp(a) were included in the analysis. † First performed follow-up measurement after initiation of PCSK9 inhibitor therapy was included in the analysis.

|  | Overall cohort | | Without diabetes mellitus | | Diabetes mellitus any type |  | T2D |  |  |
| --- | --- | --- | --- | --- | --- | --- | --- | --- | --- |
|  | HbA1c  (mmol/mol) | p-value * | HbA1c  (mmol/mol) | p-value * | HbA1c  (mmol/mol) | p-value * | HbA1c  (mmol/mol) | p-value * |  |
| Baseline | 41  (37 – 49)  (n=139) |  | 38  (36 – 40) (n=81) |  | 52 (48 – 59)  (n=58) |  | 52  (48 – 57)  (n=51) |  | |
| Month 3 | 42  (37 – 52)  (n=97) | 0.876 | 38 (36 – 40)  (n=51) | 0.204 | 52  (47 – 63) (n=46) | 0.625 | 52  (45 – 62)  (n=40) | 0.539 | |
| Month 6 | 42  (37 – 52)  (n=98) | 0.540 | 38  (36 – 40)  (n=53) | 0.054 | 53  (47 – 62)  (n=45) | 0.702 | 52  (47 – 61)  (n=40) | 0.826 | |
| Month 9 | 41  (37 – 51)  (n=78) | 0.110 | 38  (35 – 39)  (n=43) | 0.052 | 52  (47 – 57)  (n=36) | 0.333 | 51  (46 – 57)  (n=32) | 0.454 | |
| Month 12 | 42  (37 – 49)  (n=61) | 0.083 | 37  (35 – 39)  (n=31) | 0.505 | 49  (45 – 56)  (n=30) | 0.054 | 47  (43 – 57)  (n=26) | 0.215 | |

**Table S6.** HbA1c levels over time of the overall cohort, without and with diabetes mellitus at baseline. Data are median (IQR). * P-values were calculated by using the Wilcoxon signed-rank test and refers to the comparisons of laboratory data between baseline and defined time-point.
